# Reciprocal regulation of miR-1205 and E2F1 modulates progression of laryngeal squamous cell carcinoma

**DOI:** 10.1038/s41419-019-2154-4

**Published:** 2019-12-04

**Authors:** Pei Li, Xi-Jun Lin, Yang Yang, An-Kui Yang, Jin-Ming Di, Qi-Wei Jiang, Jia-Rong Huang, Meng-Ling Yuan, Zi-Hao Xing, Meng-Ning Wei, Yao Li, Xiao-Hui Yuan, Zhi Shi, Hui Liu, Jin Ye

**Affiliations:** 1 Department of Otolaryngology-Head and Neck Surgery, The Third Affiliated Hospital, Sun Yat-sen University, Guangzhou, Guangdong China; 2 Department of Cell Biology & Institute of Biomedicine, National Engineering Research Center of Genetic Medicine, Guangdong Provincial Key Laboratory of Bioengineering Medicine, College of Life Science and Technology, Jinan University, Guangzhou, Guangdong China; 3 Department of Head and Neck, Sun Yat-sen University Cancer Center; State Key Laboratory of Oncology in South China; Collaborative Innovation Center for Cancer Medicine, Guangzhou, Guangdong China; 4 Department of Urology, The Third Affiliated Hospital, Sun Yat-sen University, Guangzhou, Guangdong China; 5 Division of Pulmonary and Critical Care, Department of Internal Medicine, The Third Affiliated Hospital, Sun Yat-sen University, Guangzhou, Guangdong China

**Keywords:** Head and neck cancer, Translational research

## Abstract

The burgeoning functions of many microRNAs (miRs) have been well study in cancer. However, the level and function of miR-1205 in laryngeal squamous cell cancer remains unknown. In the current research, we validated that miR-1205 was notably downregulated in human laryngeal squamous cell carcinoma (LSCC) samples in comparison with tissues adjacent to LSCC, and correlated with T stage, lymph node metastasis, and clinical stage. Using Kaplan–Meier analysis indicates that high expression of miR-1205 has a favorable prognosis for patients with LSCC. Functional assays show that enforced miR-1205 expression attenuates the migration, growth, and invasion of LSCC cells. And E2F1 is verified to be a target of miR-1205, while E2F1 binds to miR-1205 promoter and transcriptionally inhibits miR-1205 expression. Overexpression of E2F1 reverses the inhibitory impacts of miR-1205 on LSCC cells in part. Importantly, E2F1 is abnormally increased in LSCC tissues, and its protein levels were inversely relevant to miR-1205 expression. High E2F1 protein level is in connection with clinical stage, T stage, lymph node metastasis, and poor prognosis. Consequently, reciprocal regulation of miR-1205 and E2F1 plays a crucial role in the progression of LSCC, suggesting a new miR-1205/E2F1-based clinical application for patients of LSCC.

## Introduction

Laryngeal cancer is the14th most common malignancy worldwide in the male in contrast to that rarely in the female^1^. It is approximated that 13,660 new cases and 3660 deaths caused by laryngeal cancer in the United States in 2017^1^, while 26,400 new cases and 14,500 death cases in China^2^. Laryngeal squamous cell carcinoma (LSCC) occupies ~90% of all the larynx malignant tumors^3,4^. In spite of therapeutic advances, most advanced LSCC patients have not obtained a desired outcome in the last 20 years. Thus, a profound and detailed understanding of the intrinsic molecular mechanisms underlying LSCC tumorigenesis and progression is urgently needed.

MicroRNAs (miRs) are classical endogenous noncoding RNAs that usually play crucial regulatory parts in diverse physiological and pathological progresses through a posttranscriptional mechanism by sequence-specific binding with the 3′-untranslated regions (UTRs) of target genes^5^. Studies have shown that miRs serve as crucial roles in tumorigenesis and development of cancers. In fact, miRs play the role of oncogenes or tumor suppressors by regulating their respective target genes, which are usually dysregulated in different types of cancer^6,7^. In this study, we have testified that the level of a novel miR-1205 is notable decreased in tissues and cells of LSCC, and high-level miR-1205 suppresses the migration, proliferation, and invasion of LSCC cells. Furthermore, our data have further shown that E2F1 is a direct downstream target of miR-1205 and can reverse the inhibitory effect of miR-1205 on the LSCC malignant phenotypes. E2F1 transcriptionally represses miR-1205 expression and thus forms a reciprocal regulation with miR-1205. And the expressions of miR-1205 and E2F1 protein are inversely correlated with LSCC tissues and connected with clinical stage, T stage, lymph node metastasis, and poor prognosis.

## Materials and methods

### Patients and tissue samples

From July 2008 to June 2015, 44 cases of LSCC specimens and matching adjacent tissues were required from the Department of Head and Neck of Sun Yat-sen University Cancer Center (Guangzhou, China). All patients were diagnosed as LSCC for the first time, who was performed total or partial laryngectomy without chemotherapy or neoadjuvant therapy before and after surgery. Strictly followed the 2002 International Union Against Cancer for the laryngeal cancer staging standards (clinical, endoscopic, and imaging). All signed informed consent forms were agreed from patients. This item was endorsed by the ethics committee of the Third Affiliated Hospital of Sun Yat-sen University.

### Cell culture

The cell line Hep-2, normal bronchial epithelium cell line 16HBE and embryonic kidney cell HEK293T were obtained from CCTCC, China Center for Type Culture Collection. The cell line KB-3-1 was provided by Dr. Zhesheng Chen (St. John’s university, USA). The cells were cultured at an moist atmosphere of 37 °C with 5% CO_2_ in Dulbecco’s modified Eagle’s medium (DMEM) containing 100 unit/ml penicillin, 10% fetal bovine serum (FBS), and 100 ng/ml streptomycin. Anti-E2F1 (3472) and anti-Survivin (2808) antibodies were obtained from Cell Signaling Technology. Anti-Ki-67 (2724-1) antibody was purchased from Abcam. Anti-Cyclin E (51-1459GR) antibody was from BD Pharmingen. Anti-Flag (F1804) antibody and anti-Flag (A2220) Affinity Gel were from Sigma-Aldrich. Anti-GAPDH (KM9002) antibody was from Tianjin Sungene Biotech. Anti-E2F1 (RLT1442) and anti-α-Tubulin (4777) antibodies were from Ruiying Biological.

### Plasmid

The synthesized precursor hsa-miR-1205 (Supplementary Table 1) was cloned into the lentiviral vector of pLKO.1-GFP to generate the hsa-miR-1205 lentivirus construct. The E2F1 cDNA was cloned into the lentiviral vector of LV5. Lentivirus was transfected in HEK293T cells and the supernatant of the medium was harvested after 48 h. And the lentivirus was infected into Hep-2 and KB-3-1 cells to established stable cell lines, followed by puromycin selection.

### RNA extraction and real-time quantitative polymerase chain reaction

Total RNA of tissues and cells was extracted using HiPure Total RNA Mini Kit (Magen). Reverse transcription was carried out with HiFi-script cDNA kit (Cwbio) in the light of the manufacturer’s instruction. The Bestar^TM^ Real time PCR Master Mix was applied to real-time quantitative polymerase chain reaction (RT-qPCR) by SYER Green Method. All reactions were performed on an ABI 7900HT instrument (Applied Biosystems). All experiments were implemented at three independent times and all reactions were performed in triplicate at least. The dates of RT-qPCR were normalized to U6 using the 2^−ΔΔCt^ method. The primers were ordered from Sangon Biotech. The mentioned primer sequences are exhibited in Supplementary Table 1.

### Western blot analysis

In brief, cells were collected and proteins were extracted in radioimmunoprecipitation assay buffer containing 0.03% aprotinin, 10 ng/ml phenylmethylsulfonyl fluoride, 1 μM sodium orthovanadate, 0.1% sodium dodecyl sulfate (SDS), and 0.5% sodium deoxycholate at 4 °C for 30 min. The lysates were centrifuged at 14,000 *g* for 10 min, and the supernatants were harvested and stored at −80 °C. Protein were isolated in 12% SDS–PAGE gels and transferred onto the polyvinylidene difluoride membrane. After that, the membrane was blocked by 5% bovine serum albumin (BSA), and then hatched with the relative antibody and secondary antibody, successively. GAPDH or α-Tubulin was appplied to a loading control. According to instruction, signal was measured through the chemiluminescent gel imaging system (Bio-RAD).

### Immunohistochemistry assay

In brief, tissues and subcutaneous tumors of human LSCC were fixed in paraformaldehyde and washed with phosphate-buffered saline (PBS), then embedded in paraffin, and tumor tissues were stained with antibodies. The protein expression was quantified through the following formula: Immunohistochemical score = percentage of positive cells × intensity score. The staining intensity was evaluated as follows: 0, negative (no staining); 1, weak (light yellow); 2, moderate (yellow brown); and 3, intense (brown).

### MTT assay

MTT (3-(4,5-dimethyl-2-thiazolyl)-2,5-diphenyl-2-H-tetrazolium bromide) assay was applied to validate cell proliferation. In brief, cells were cultured in a 96-well plate and treated with different concentrations of agents. After 72 h, 0.5 mg/ml MTT was added to each well and incubated for 4 h. After that, the MTT solution and medium were removed, and 100 μl DMSO was added to dissolved formazan crystals in each well. Multiscan Spectrum (Thermofisher) was used to measure the absorbance at 570 nm.

### Sphere formation assay

Cells were digested by trypsin and suspended in medium containing 10% FBS and 0.3% agar, and then plated in 12-well plate at 5 × 10^2^ cells/well density. The agar cell mixture was coated with 0.5% agar. The cells were cultured at a moist atmosphere containing moderate fresh medium. After 2 weeks, each well was examined with an optical microscope and the total number of spherical colonies was calculated. The size of spherical cells was measured and compared with wild-type cells.

### Wound healing assay

In brief, cells (5 × 10^5^ per well) were cultivated at 6-well dishes. Till the cell fusion arrive in 80–90%, 10 μl sterilized pipette tip was used to scrap the cell monolayer and then washed the cells with PBS twice. After that, cells were incubated in serum-free medium for 24 or 48 h, and the scratch were observed and captured at appointed time. The lengths of gap were measured by the photomicrographs.

### Transwell assay

A modified Boyden chamber (Corning) containing matrigel-coated polycarbonate membrane filter was used to perform invasion test. Cells and the serum-free medium were added in the upper compartment of chamber, and medium containing 10% FBS were plated to the lower chamber, and allowed to culture under the condition of 37 °C and 5% CO_2_ for 24 h. Then cells on the upper layer of the membrane were wiped off, and cells invaded to the bottom surface were photographed and counted.

### Luciferase reporter assay

The E2F1 3′UTRs reporter or miR-1205 promoter reporter vectors were transfected into Hep-2, KB-3-1, or HEK293T cells. After 24 h, cell lysates were harvested and the Dual Luciferase Reporter Assay Kit (Promega) was applied to detect the Firefly/Renilla luciferase activity.

### Chromatin immunoprecipitation assay

Cells from 1% formaldehyde cross-linked for 10 min were sheared to a fragment range of 100–500 bp by sonication in a crushed ice bath with eight 5-s bursts of 270 W, with a 30 s interval between bursts using an ultrasonic processor (SCIENTZ, JY92-IIDN). Precleared chromatin was incubated overnight on a rotating platform at 4 °C with anti-Flag affinity gel. Precipitated chromatin complexes were eluted with elution buffer (0.1 M NaHCO3 and 1% SDS) and treated with 10 μl 0.5 M EDTA, 20 μl 1 M Tris, and 1 μl proteinase K to reverse the cross-link. DNA was purified by DNA purification Kit (Promega, USA). Finally, purified DNA was analyzed by PCR to measure enrichment of DNA fragments in the putative E2F1-binding sites 2 of the miR-1205 promoter by using two pairs of relative primers, containing the region between −391 bp and −191bp of the miR-1205. Two pairs of PCR primers for detecting the putative-binding site 2 (BS2) of the miR-1205 promoter are listed at Supplementary Table 1. Chromatin immunoprecipitation (CHIP)-PCR products were isolated by electrophoresis through 2% agarose gels in 110 V voltage for 20 min, and visualized after staining with ethidium bromide.

### Nude mice tumorigenesis assay

Balb/c mice were acquired from Shanghai SLAC Laboratory Animal Co. Five female nude mice were used randomly for per group, age of 5 weeks weighing 16–18 g. Hep-2 cells or KB-3-1 cells, infected with control or has-miR-1205 lentivirus, were suspended 4 × 10^6^ in 100 μl of DMEM respectively and were injected into the shoulder of the female nude mouse for each mouse. The two perpendicular diameters (a and b) and animals body weights of tumor were recorded blindingly every 5 days. The following formula was used to calculate the tumor volume (V):

$${\mathrm{V}} = \frac{\pi }{6}\left( {\frac{{a + b}}{2}} \right)^3$$

All experimental procedures were approved by the Institutional Animal Care and Use Committee of Sun Yat-Sen University.

### Statistical analysis

The version SPSS 20.0 software was used to calculate statistical analysis. Data were exhibited as mean ± s.d. or median with the interquartile range. The Student’s *t*-test and Mann–Whitney *U*-test were applied to analyze comparisons between two groups, and one-way analysis of variance (ANOVA) and Kruskal–Wallis test were employed to analyze comparisons among three groups. The relationship between the expression of miR-1205 and E2F1 protein were accessed by Pearson’s correlation. The tumor volume was calculated by repeated measures analysis of variance. The prognostic ability was evaluated by receiver operating characteristic (ROC) curve analyses. The survival rates of patients were conducted using Kaplan–Meier analysis and the log-rank test. Data were considered statistically significant when *P* < 0.05.

## Results

### Downregulation of miR-1205 in LSCC is related to clinical stages, T stages, lymph node metastasis, and poor prognosis

**Fig. 1:**
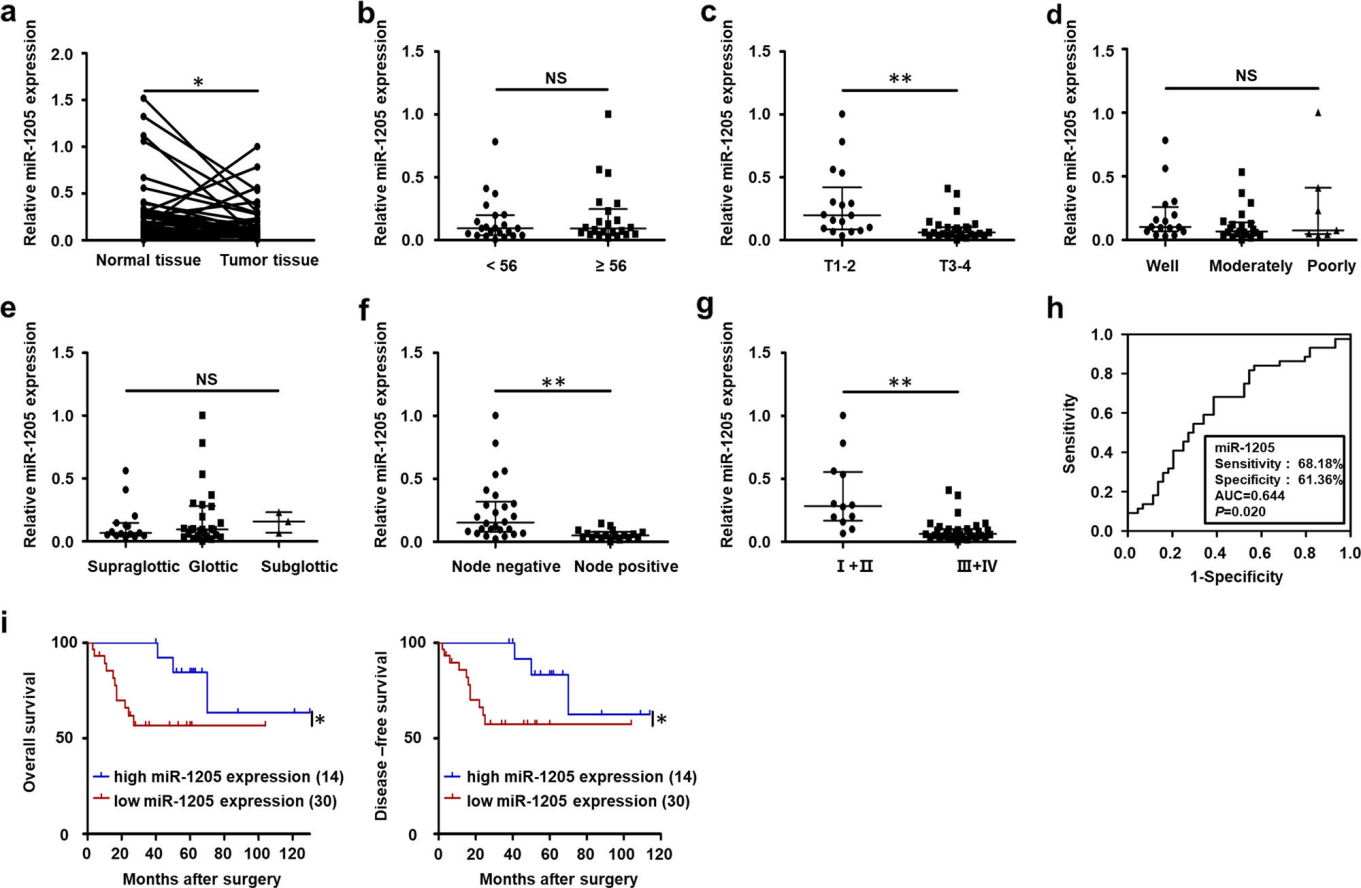
Downregulation of miR-1205 in LSCC is correlated with T stages, lymph node metastasis, clinical stages, recurrence, and poor prognosis. **a** RT-qPCR analysis of the relative miR-1205 expression in 44 pairs of LSCC tissues and adjacent normal tissues with Student’s *t*-test. The relative miR-1205 expression in two groups of LSCC tissues classified by age **b**, T stage **c**, lymph node metastasis **f**, and clinical stage **g** were analyzed with Mann–Whitney *U*-test. The relative miR-1205 expression in three groups of LSCC tissues classified by differentiation **d** and primary location **e** were analyzed with Kruskal–Wallis test. **h** ROC curve analysis of the discrimination between LSCC tissues and adjacent normal tissues by miR-1205. **i** Kaplan–Meier analysis of overall survival and disease-free survival curves for LSCC patients with high and low expression of miR-1205. Data are presented as mean with the interquartile range; *P < 0.05; **P < 0.01; NS, no statistical significance.

To explore the level of miR-1205 of LSCC tissues, a total 44 cases of LSCC samples and matching adjacent tissues were examined with RT-qPCR. The level of miR-1205 was significantly decreased in LSCC, with 68% (30/44) of the tumor tissues showing reduced expression compared to matched normal controls (Fig. 1a). Moreover, we found that the level of miR-1205 was relevant to T stage (Fig. 1c), lymph node metastasis (Fig. 1f), and clinical stage (Fig. 1g), other than age (Fig. 1b), tumor differentiation (Fig. 1d) and primary location (Fig. 1e; Supplementary Table 2). The level of miR-1205 in stage T1-2, I + II, and negative lymph node metastasis groups were higher than that in stage T3-4, III + IV, and positive lymph node metastasis groups, respectively (Fig. 1c, g, f). To further evaluate the diagnostic value of miR-1205 for LSCC, the ROC analysis was conducted. As shown in Fig. 1h, the expression of miR-1205 could be used as an important parameter to differentiate LSCC and corresponding normal tissues with an range under the ROC curve of 0.644 (sensitivity = 68.18%, specificity = 61.36%; *P* = 0.020). Furthermore, Kaplan–Meier survival analysis of LSCC patients indicated that low miR-1205 level was in connection with poor overall survival as well as disease-free survival (Fig. 1i). In brief, these findings indicate that downregulation of miR-1205 in LSCC tissues is related to clinical stages, T stages, lymph node metastasis, and poor prognosis.

### MiR-1205 suppresses the growth, migration, and invasion of LSCC cells in vitro

**Fig. 2:**
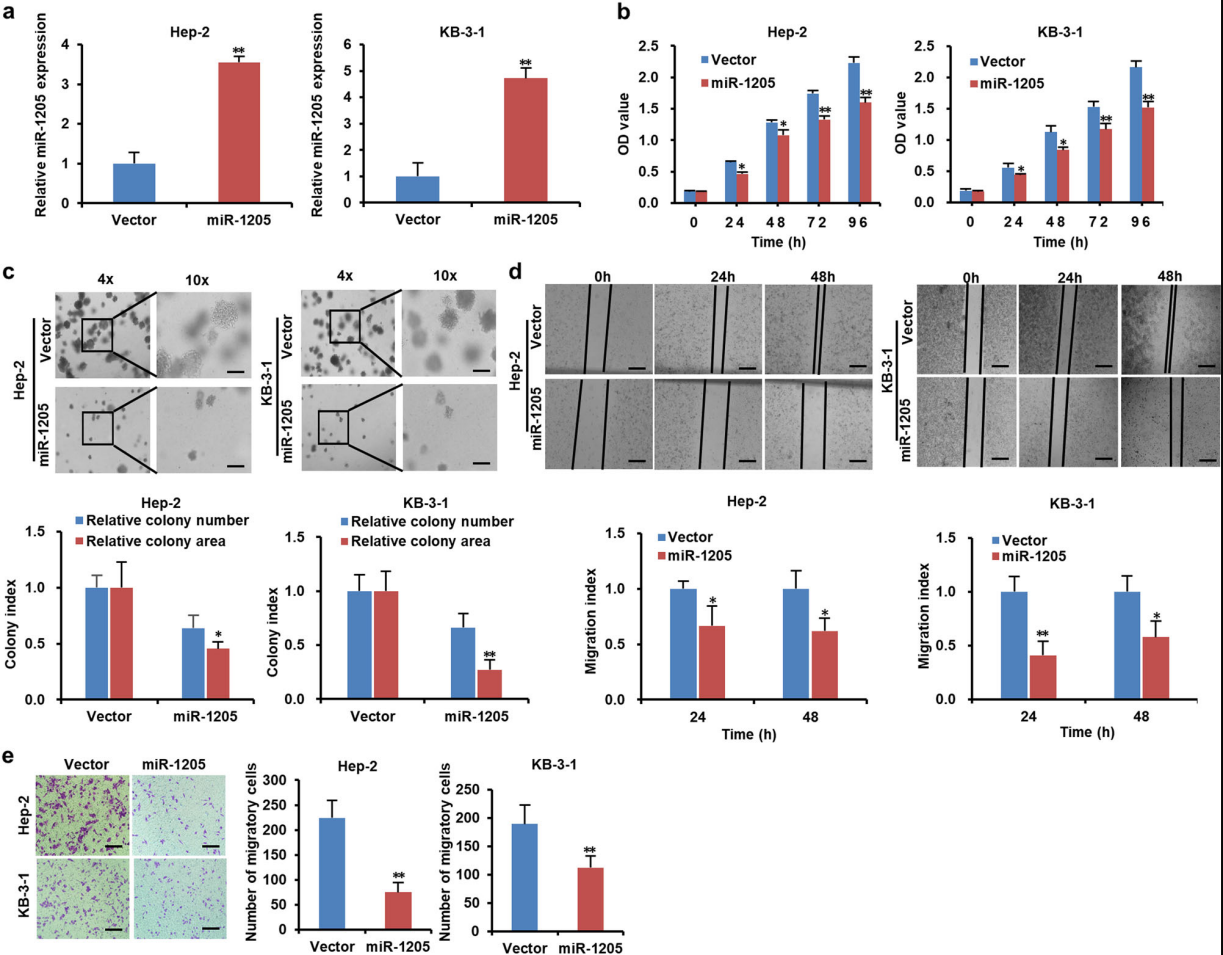
MiR-1205 suppresses the growth, migration, and invasion of LSCC cells. **a** RT-qPCR analysis of the relative miR-1205 expression in Hep-2 and KB-3-1 cells expressed vector control and miR-1205. **b** Cell proliferation of the indicated cells as determined with MTT assay. Representative images and quantification of the indicated cells sphere as determined with sphere formation assay. Scale bar, 200 μm **c**, cells migration as determined with wound healing assay **d** and cells invasion as determined with transwell assay **e**. Data are presented as mean ± . Student’s *t*-test was used for statistical analysis; *P < 0.05; **P < 0.01.

To explore the function of miR-1205 in LSCC, we firstly determined the level of miR-1205 in normal human bronchial epithelial cell line 16HBE and two LSCC cell lines Hep-2 and KB-3-1. As shown in Supplementary Fig. 1a, the expressions of miR-1205 in Hep-2 and KB-3-1 cells were dramatically lower than those in 16HBE. Next, Hep-2 and KB-3-1 cells were infected with lentivirus expressing precursor miR-1205, which successfully upregulated miR-1205 in the cells (Fig. 2a). Overexpression of miR-1205 significantly attenuated the cell proliferation in Hep-2 and KB-3-1 cells (Fig. 2b). In addition, miR-1205-transduced Hep-2 and KB-3-1 cells formed smaller and less spheres than vector control cells (Fig. 2c). Analysis from the transwell assay and wound healing indicated that the invasion and migration abilities of miR-1205-overexpressing cells were dramatically decreased when compared with control cells (Fig. 2d, e). In general, our data suggest that miR-1205 may function as a inhibitory gene in LSCC cells.

### MiR-1205 inhibited the growth of LSCC cells in vivo

**Fig. 3:**
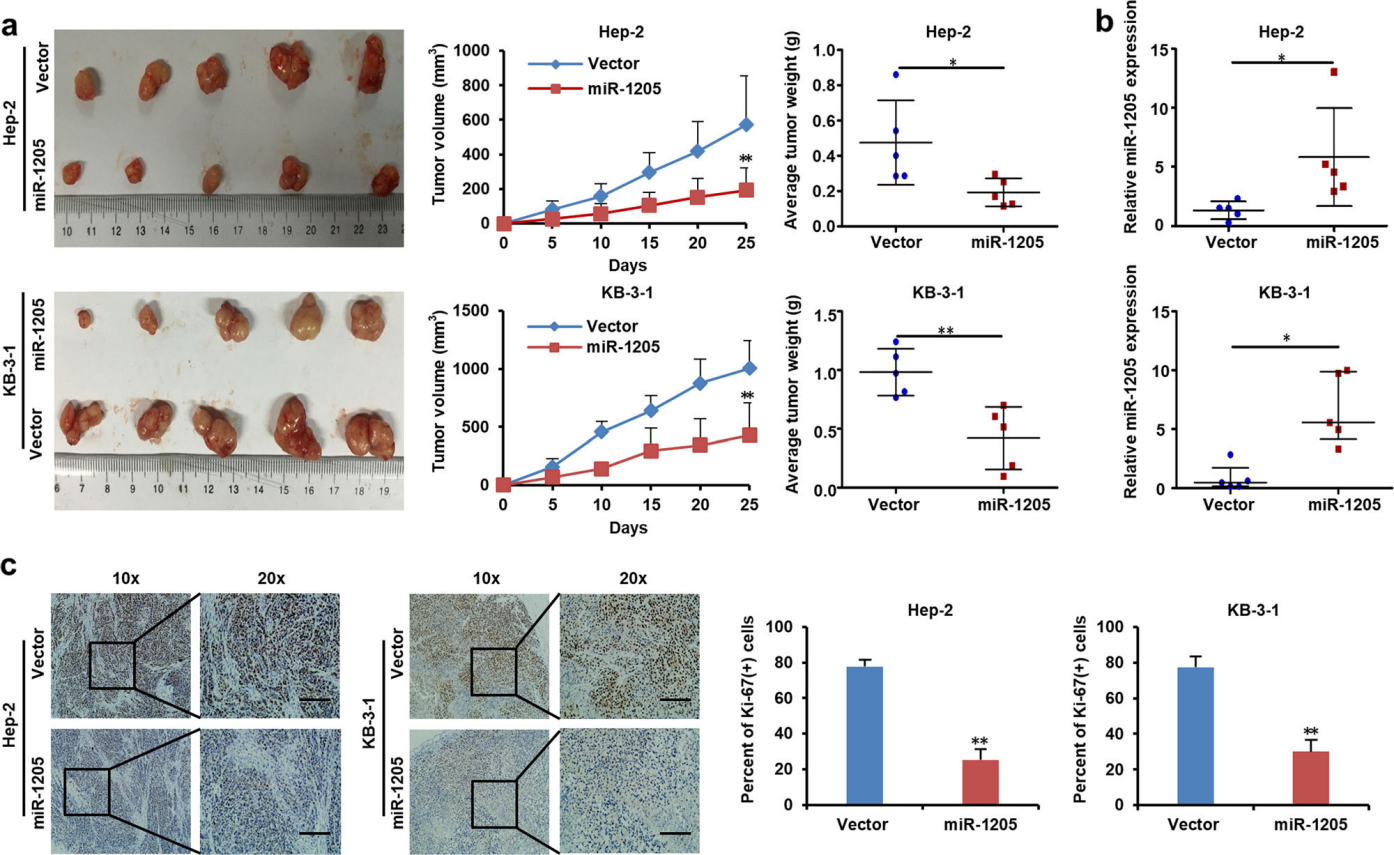
MiR-1205 inhibits the growth of LSCC cells in vivo. **a** The subcutaneous tumors of the indicated cells. The tumor volume and weight were determined by repeated measures analysis. **b** RT-qPCR analysis of the relative miR-1205 expression in the subcutaneous tumors of the indicated cells. **c** Representative images and quantification of Ki-67^+^ cells in the indicated tumors as determined with IHC assay. Scale bar, 100 μm. Data are presented as mean ± s.d. Student’s *t*-test was used for statistical analysis; *P < 0.05; **P < 0.01.

To further inquire the impact of miR-1205 on LSCC cells in vivo, we established subcutaneous tumor model in nude mice bearing Hep-2 and KB-3-1 xenografts. As shown in Fig. 3a, overexpression of miR-1205 notably suppressed the proliferation of Hep-2 and KB-3-1 xenografts by decreasing the tumor volumes and weights. RT-qPCR analysis confirmed that the expression of miR-1205 was continuously higher in miR-1205-transduced Hep-2 and KB-3-1 tumors than vector control (Fig. 3b). Immunohistochemistry (IHC) staining results showed that miR-1205 overexpression prominently decreased the numbers of Ki67^+^ proliferating cells in Hep-2 and KB-3-1 tumors (Fig. 3c), implying that miR-1205 not only attenuates cell proliferation but also suppresses angiogenesis in tumor. Collectively, these discoveries demonstrated that miR-1205 may be a tumor inhibitor in vivo.

### MiR-1205 directly targets the 3’UTR of E2F1

**Fig. 4:**
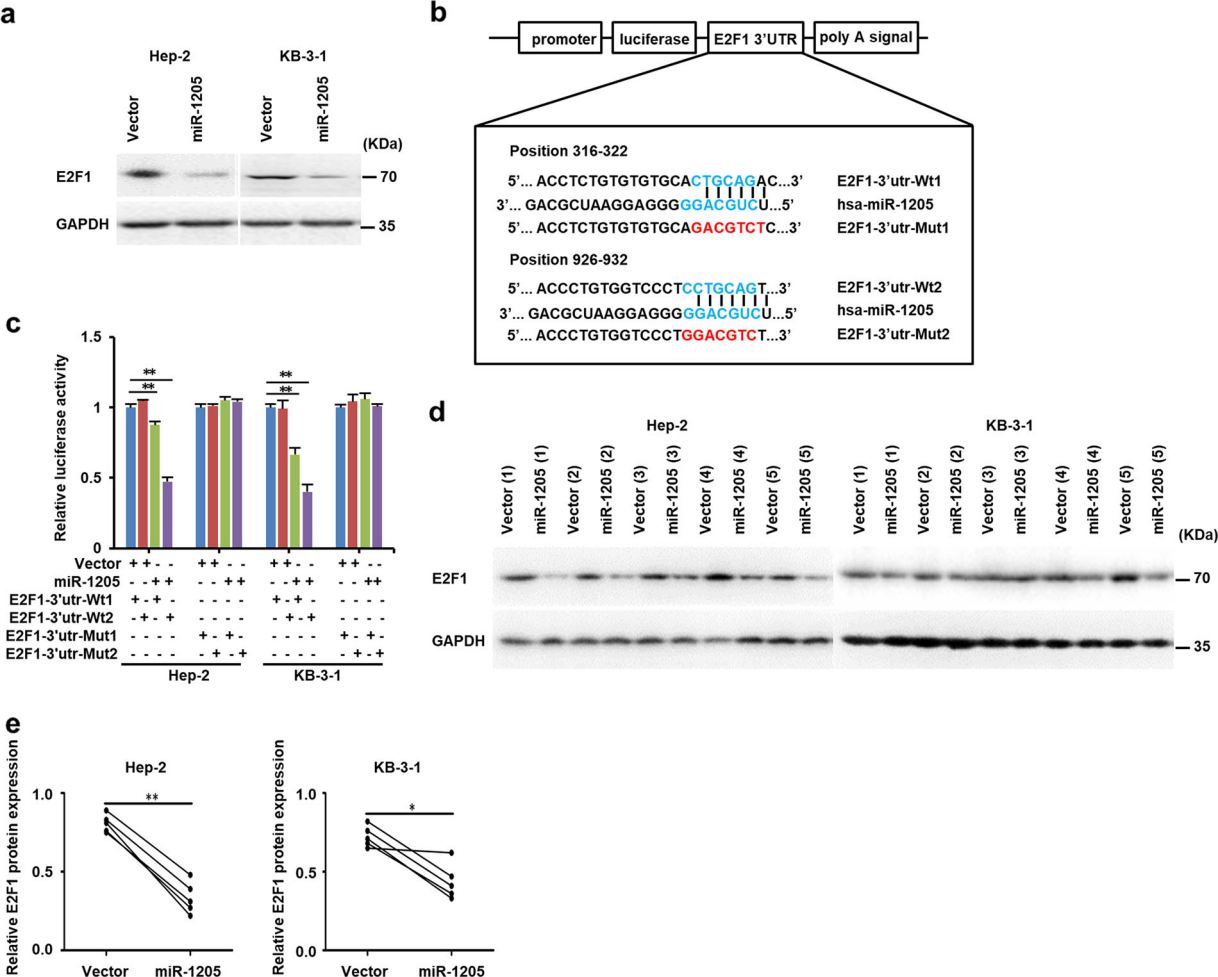
MiR-1205 directly targets E2F1. **a** Western blot analysis of E2F1 protein expressions in the indicated cells. **b** A schematic diagram of the reporter constructs showed the wild type (Wt) and mutant (Mut) sequences of the miR-1205-binding sites within human E2F1 3′-UTR. **c** Luciferase activity of reporters with E2F1 Wt or Mut 3′-UTR in the indicated cells. **d**, **e** Western blot analysis and quantification of E2F1 protein expression in the tumors of the indicated cells. GAPDH was the loading control. Data are presented as mean ± s.d. Student’s *t*-test was used for statistical analysis; **P < 0.01.

To understand the mechanism of miR-1205 as a tumor inhibitor in LSCC, we used three bioanalysis algorithms, including TargetScan, miRanda, and PITA, to verified the targets of miR-1205. All three analyses indicated that E2F1 is a downstream target of miR-1205. Moreover, overexpression of miR-1205 in Hep-2 and KB-3-1 cells distinctly decreased the E2F1 protein levels (Fig. 4a). The predicted interactive sites between miR-1205 and E2F1 3′UTR are illustrated in Fig. 4b. Then luciferase assay was used to examine whether there was a direct reciprocity between miR-1205 and E2F1. As exhibited in Fig. 4c, increasing the levels of miR-1205 significantly attenuated the luciferase activity of wild-type E2F1 3′UTR region in Hep-2 and KB-3-1 cells, but not mutant 3′UTR. The results suggesting that the predicted binding sites between miR-1205 andE2F1 are responsible for this miRNA–mRNA interaction (Supplementary Fig. 1b). Furthermore, analysis of xenograft in mice revealed that the E2F1 level in miR-1205-transfected tumors were notable lower than those in control groups (Fig. 4d, e). Altogether, our findings support that miR-1205 suppresses E2F1 expression through targeting its 3′UTR.

### E2F1 directly represses the expression of miR-1205

**Fig. 5:**
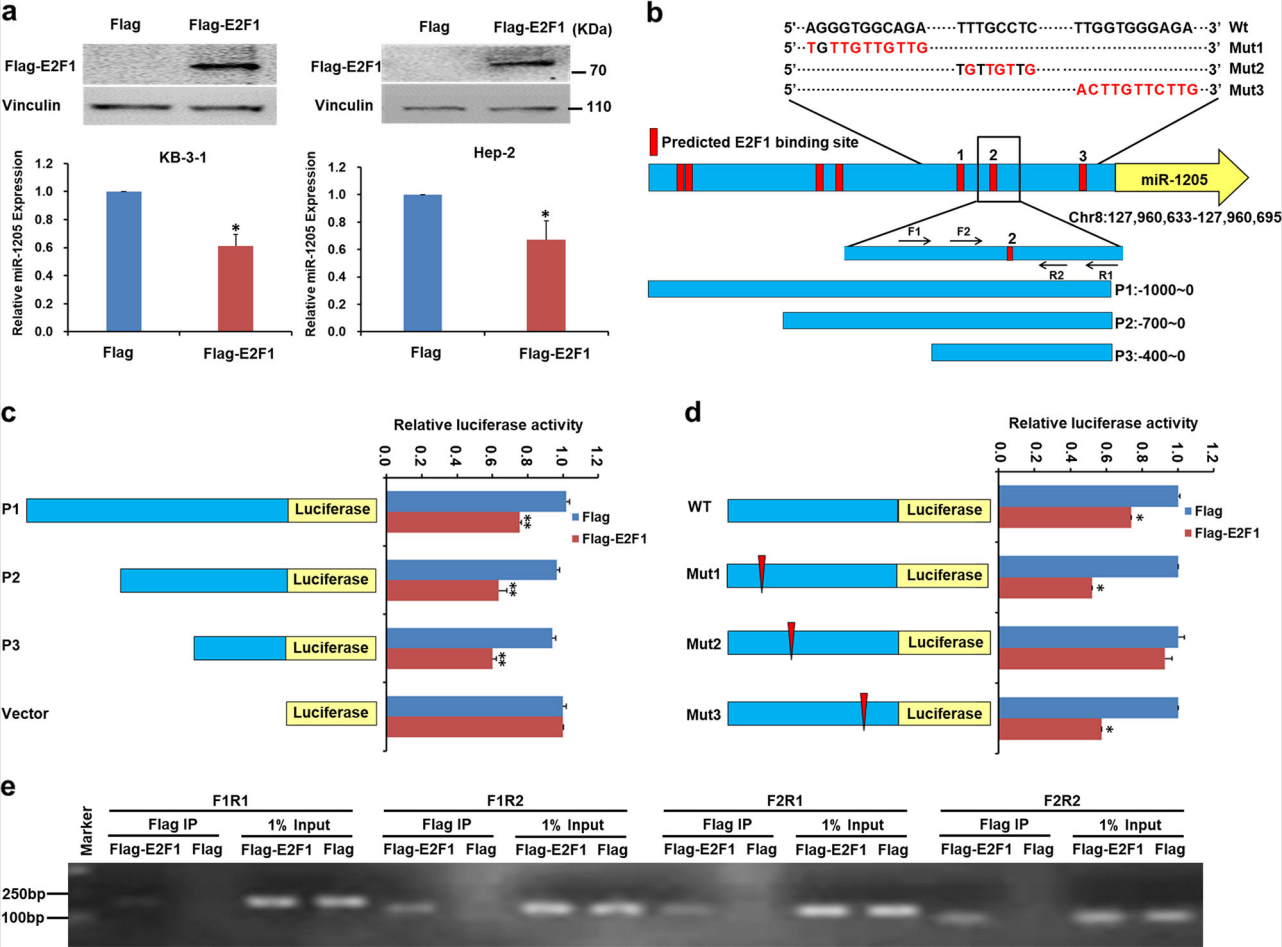
E2F1 directly represses the expression of miR-1205. **a** RT-qPCR analysis of the relative miR-1205 expression in Hep-2 and KB-3-1 cells expresses Flag and Flag-E2F1. **b** Schematic representation of the miR-1205 locus on chromosome 8. The red squares indicated seven putative E2F1-binding sites, while the yellow arrow indicated transcription start sites. Three blue bars indicated truncated promoter fragments, respectively. Three mutations of binding sites were Mut1, Mut2, and Mut3. The contents of the amplification represent different mutation sites, and the red bases stand for the mutated bases. The F1, F2, R1, and R2 arrows represented different ChIP-PCR primers of BS2. **c**, **d** HEK293T cells were transfected with a luciferase reporter construct containing the indicated genomic regions and mutants with or without Flag-E2F1 plasmids. Relative luciferase activities were measured 24 h after transfection. The results are depicted as the ratio of Firefly luciferase and Renilla luciferase activity. **e** PCR gel showing E2F1 was enriched in the binding site 2 (BS2) of the putative miR-1205 promoter by ChIP assay. Data are presented as mean ± s.d. Student’s *t*-test was used for statistical analysis; *P < 0.05, **P < 0.01.

Since E2F1 is a transcription factor, we evaluated whether E2F1 would in turn regulate the expression of miR-1205. To examine the role of E2F1 in regulating miR-1205, we first used eukaryotic expression plasmid to increase E2F1 expression in Hep-2 and KB-3-1 cells. Next, qPCR analysis indicated that miR-1205 expression was markedly decreased in both cells after E2F1 overexpression, suggesting that E2F1 is an upstream regulator of miR-1205 (Fig. 5a). To test the transcriptional regulatory mechanisms of miR-1205 expression, Ensembl (http://asia.ensembl.org/index.html) was used to search a 1 kb region upstream of the transcription start site of miR-1205. We then input the miR-1205 promoter sequences into JASPAR (http://jaspar.binf.ku.dk/), a transcription factor prediction website, to predict the binding sites of E2F1. Seven E2F1-binding motifs were predicted inside the putative miR-1205 promoter region (Fig. 5b). Accordingly, the 1 kb genomic fragment upstream of miR-1205 (P1) containing seven E2F1-binding sites was cloned and truncated from −700 to 0 bp (P2) and −400 to 0 bp (P3), to construct the promoter vector according to the predicted binding site (Fig. 5b). Luciferase activities were measured after transfected with or without E2F1 expression. As illustrated in Fig. 5c, cells transfected with E2F1 in all three reporters (P1, P2, and P3) exhibited the parallel effect of suppressing the luciferase activity compared with empty vectors. Therefore, we focused on the three predicted sites in −400–0 bp and constructed their reporter vectors with three E2F1 putative-binding sites (Mut1, Mut2, and Mut3). Followed by a dual-luciferase experiments, Mut1 and Mut3 did not change the effect of decreasing in E2F1-mediated luciferase activity but Mut2 did (Fig. 5d), which indicates that E2F1 binds to the BS2 of miR-1205 promoter. Furthermore, ChIP assays verified that E2F1 was enriched in the BS2 of miR-1205 promoter in HEK293T cell (Fig. 5e). Altogether, these discoveries provided convincing sight that E2F1 bind to the miR-1205 promoter and it attenuated the transcription of miR-1205 in a competitive relationship to repress each other during the evolution of tumors.

### E2F1 partially reverses the suppressive impacts of miR-1205 on the growth, migration, and invasion of LSCC cells

**Fig. 6:**
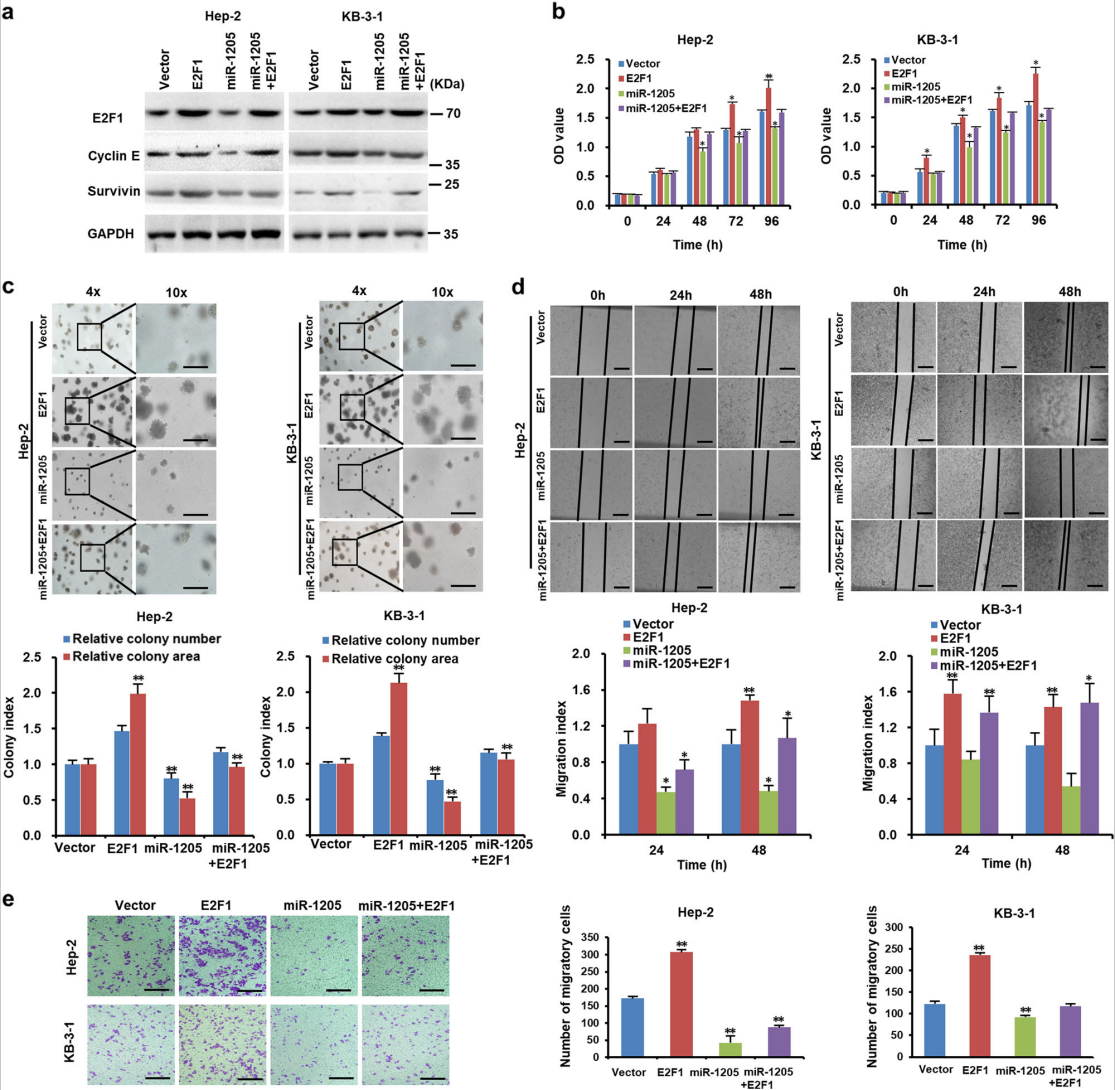
E2F1 partially reverses the suppressive effects of miR-1205 on the growth, migration, and invasion of LSCC cells. **a** Western blot analysis of E2F1, cyclin E, and survivin protein expressions in the indicated cells. GAPDH is the loading control. **b** Cell proliferation of the indicated cells as determined with MTT assay. Representative images and quantification of the indicated cells sphere as determined with sphere formation assay. Scale bar, 200 μm **c**, cells migration as determined with wound healing assay **d** and cells invasion as determined with transwell assay **e**. Data are presented as mean ± SD. Student’s *t*-test was used for statistical analysis; *P < 0.05; **P < 0.01.

To verify the role of E2F1 in miR-1205 biological function, miR-1205-overexperssing cells were transduced with lentivirus expressing E2F1 gene, which successfully upregulated the protein expressions of E2F1 and its targets cyclin E and survivin in the cells (Fig. 6a). Promotion of cell proliferation by ectopic expression of E2F1 in the vector control cells was more significant than in the miR-1205-overexperssing cells (Fig. 6b). In addition, ectopic expression of E2F1 improved the formation of larger and more spheres in the vector control cells than in the miR-1205-overexperssing cells (Fig. 6c). The migration and invasion abilities of vector control cells by increasing E2F1 were enhanced more dramatically than those of miR-1205-overexpressing cells control cells (Fig. 6d, e). Therefore, these data suggest that restored expression of E2F1 partially reverses the suppressive impacts of miR-1205 on the growth, migration, and invasion of LSCC cells.

### Upregulation of E2F1 protein in LSCC is related to clinical stages, T stages, lymph node metastasis, and poor prognosis

**Fig. 7:**
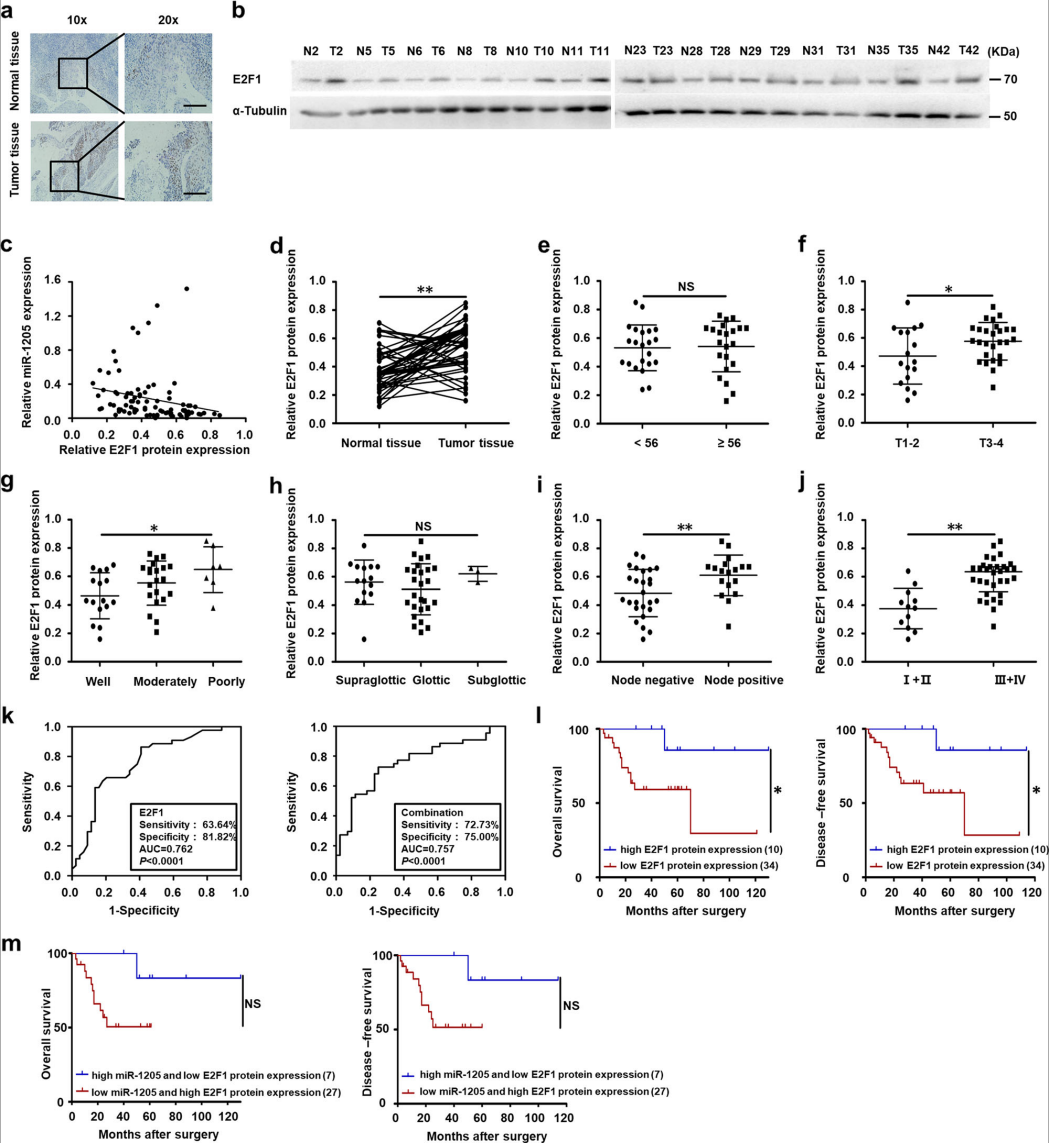
Upregulation of E2F1 protein in LSCC is correlated with miR-1205, T stages, lymph node metastasis, clinical stages, and poor prognosis. Representative results of IHC **a** and Western blot analysis **b** showed the expression of E2F1 protein in LSCC tissues and adjacent normal tissues. Scale bar, 100 μm. **c** Pearson’s correlation scatter plot of the expressions of miR-1205 and E2F1 protein in human LSCC tissues. **d** Analysis of the E2F1 protein expression in LSCC tissues and adjacent normal tissues with Student’s *t*-test. The E2F1 protein expression in two groups of LSCC tissues classified by age **e**, T stage **f**, lymph node metastasis **i**, and clinical stage **j** were analyzed with Student’s *t*-test. The E2F1 protein expression in three groups of LSCC tissues classified by differentiation **g** and primary location **h** were analyzed with one-way ANOVA. **k** ROC curve analysis of the discrimination between LSCC tissues and adjacent normal tissues by E2F1 or combination of miR-1205 and E2F1. **l**, **m** Kaplan–Meier analysis of overall survival and disease-free survival curves for LSCC patients with high or/and low expression of miR-1205. Data are presented as mean ± s.d. *P < 0.05; **P < 0.01; NS, no statistical significance.

To further determine the clinical association with miR-1205 and E2F1, the level of E2F1 protein was also detected in the 44 cases of LSCC specimens and corresponding adjacent normal tissues. IHC and Western blot analysis indicated that the E2F1 level was higher in LSCC than corresponding normal samples (Fig. 7a, b). Moreover, the level of E2F1 protein and miR-1205 appeared a close inverse correlation in tissues (Supplementary Table 3 and Fig. 7c). Moreover, statistical analysis indicates that the level of E2F1 was related to T stage (Fig. 7f), tumor differentiation (Fig. 7g), lymph node metastasis (Fig. 7i), and clinical stage (Fig. 7j), rather than age (Fig. 7e) and tumor primary locations (Fig. 7h) (Supplementary Table 2). The level of E2F1 protein in stage T1–2, well differentiation, negative lymph node metastasis, and I + II groups were lower than that in stage T3–4, poorly differentiation, positive lymph node metastasis, and III + IV groups, respectively (Fig. 7f, g, i–j). The E2F1 protein expression could be a markedly index to differentiate LSCC tissues and adjacent normal tissues with the ROC curve of 0.762 (sensitivity = 63.64%, specificity = 81.82%; *P* < 0.0001; Fig. 7k). The combination of miR-1205 and E2F1 protein might have higher diagnostic potential with the ROC curve of 0.757 (sensitivity = 72.73%, specificity = 75.00%; *P* < 0.0001; Fig. 7k). Furthermore, Kaplan–Meier method about the patients’ survival time showed that high E2F1 level was relevant to poor overall survival, rather than disease-free survival (Fig. 7l). Patients with high miR-1205 and low E2F1 had predisposition to a favorable survival, although there was no statistically significance due to probably insufficient quantity of cases (Fig. 7m). Consequently, our results show that E2F1 may have the oncogenic function inversely with miR-1205 and potential clinical application in LSCC.

## Discussion

An increasing amount of evidence indicates that miRs can be potential diagnostic and prognostic markers for various cancers^8,9^. In our studies, we have validated that the level of miR-1205 is signally lower in clinical LSCC samples in comparison with corresponding adjacent tissues, and related to clinical stages, T stages, and lymph node metastasis. Kaplan–Meier survival analysis indicates that high miR-1205 predicts a better prognosis for LSCC patients. Functional experiments show that increasing miR-1205 expression attenuates the growth, migration, and invasion of LSCC cells, suggesting that miR-1205 may display as a tumor inhibitor in LSCC. The expression and function of miR-1205 in cancer are largely unknown until recently. It was reported MiR-1205 can be sponged by hsa_circ_0039411, hsa_circ_0034642, hsa_circ_0002052, has_circ-POSTN, and hsa_circMAN2B2 in various cancers to regulate tumorigenesis and progression^10–15^. Moreover, miR-1205 could act as a tumor suppressor in nonsmall cell lung cancer by disconnecting the synergy between KRAS and MDM4/E2F1^16^. In contrast, by targeting EGLN3 and APC2, miR-1205 has an oncogenic role in castration-resistant prostate cancer and lung adenocarcinoma, respectively^17,18^. Data above mentioned suggests that miR-1205 has emerged as a vital member involved in tumor progression.

Several other miRs have been reported as tumor suppressors or oncogenes in LSCC. For example, we recently have verified that miR-194 is notably decreased in LSCC tissues, and display as a tumor depressor in LSCC by targeting Wee1^19^. MiR-101 induces LSCC cells apoptosis via targeting CDK8, and is dramatically reduced in LSCC tissues and related to clinical stages, T stages, lymph node metastasis, and poor prognosis^20^. MiR-34a/c inhibits LSCC cells proliferation by causing cell cycle arrest via directly suppressing GALNT7, and is also decreased in LSCC tissues and related to histological differentiation and prognosis^21^. The level of miR-375 is frequently decreased in LSCC samples, and overexpression of miR-375 suppressed LSCC cell proliferation, motility and invasion by targeting IGF1R^22^. On the contrary, miR-155 functions as an oncogene to enhance the invasion and proliferation of LSCC cells via targeting SOCS1 and STAT3, and is clearly increased in LSCC tissues and correlated with differentiation and T stage^23^. MiR-93 can promote LSCC cells proliferation, migration, and invasion through directly inhibiting cyclin G2, and is also upregulated in LSCC tissues^24^. The expression of miR-27a is usually raised in LSCC tissues, and overexpression of miR-27a accelerates LSCC cells proliferation by targeting PLK2^25^.

E2F1, belonging to the family of E2F transcriptional factors, is known to regulate many genes involved in proliferation, migration, invasion, apoptosis, autophagy, senescence, and so on. Deregulated E2F1 expressions have been found in many types of cancers, including lung, esophageal, gastric, colon, pancreatic, breast and ovarian cancer, etc.^26^. It has reported that E2F1 could promote invasion and migration of prostate cancer and osteosarcoma cells through regulating CD147 and DDR1 expression^27,28^. However, the expression and role of E2F1 in LSCC remains elusive. In our study, E2F1 is upregulated in LSCC specimens, and the E2F1 level is connection with T stage, lymph node metastasis, clinical stage, and prognosis. E2F1 has been confirmed to be directly targeted by a plethora of miRs, such as miR-136, miR-302b, miR-320, miR-342-3p, miR-372, miR-622, and so on in various cancers^29–35^. In addition, previous researches have showed that E2F1 is able to transcriptionally regulate several miRs, including elevating miR-203, miR-15/16, miR-17–92 cluster, and miR-449a/b levels, and inhibiting miR-30b expression^36–40^. In this study, we identified that E2F1 is not only a direct target of miR-1205 but also directly transcriptionally represses miR-1205 expression. Furthermore, the expression of E2F1 protein and miR-1205 in LSCC tissues appeared a close inverse correlation. Most LSCCs are diagnosed at advance stage due to lack of highly sensitive and specific diagnostic biomarkers. Accumulated evidences have demonstrated miRs may be diagnostic biomarkers for LSCC. It has reported that miR-21 and miR-375 are upregulated and downregulated, respectively, in LSCC tissues, and the ratio of miR-21/miR-375 displays as a diagnostic potential with a high sensitivity and specificity to discriminate LSCC and normal tissues^41^. Deregulated miR-657 and miR-1287 in LSCC tissues are also highly sensitive, and specific for classifying LSCC and normal esophageal mucosa tissues^42^. The expression of serum exosomal miR-21 and HOTAIR is significantly higher in patients with LSCC, and the combination of miR-21 and HOTAIR is highly sensitive and specific in differentiating LSCC from benign laryngeal disease^43^. In this study, we have found that either miR-1205 or E2F1 can be a notable index to differentiate LSCC and adjacent normal samples, with practical sensitivity and specificity. Moreover, the combination of miR-1205 and E2F1 protein may have higher diagnostic potential compared with each alone. The diagnostic potential of miR-1205 and E2F1 protein needs to be validated in the larger number of serum and tissue samples in the future.

In conclusion, our results indicate a potential inhibitory function of miR-1205 in LSCC by directly targeting E2F1, while E2F1 transcriptionally represses miR-1205. The clinical data suggest that miR-1205 and E2F1 loop maybe identified as a potential diagnostic and prognostic biomarker for LSCC. Our research provides novel sights into the function of miR-1205/E2F1 reciprocal regulation in LSCC (Supplementary Fig. 2), and indicates a newmiR-1205/E2F1-based clinical application for LSCC patients.

## Supplementary information

Table S1

Table S2

Table S3

Supplementary Figure Legends

Fig S1

Fig S2
