# Supplementary material for: Reciprocal regulation of miR-1205 and E2F1 modulates progression of laryngeal squamous cell carcinoma
Source: Cell Death Dis. 2019 Dec 4;10(12):916. doi: 10.1038/s41419-019-2154-4 (PMC6893029; doi:10.1038/s41419-019-2154-4)
Supplement: Supplementary file 1 — Table S1 [file 41419_2019_2154_MOESM1_ESM.docx]

Table S1. Primers for reverse transcription and quantitative real-time PCR.

| Primers | Sequences (From 5’ to 3’) |
| --- | --- |
| U6-F | GCGCGTCGTGAAGCGTTC |
| U6-R | GTGCAGGGTCCGAGGT |
| U6-RT | GTCGTATCCAGTGCAGGGTCCGAGGTATTCGCACTGGATACGACAAAATA |
| hsa-miR-1205-F | CACGCATCTGCAGGGTTT |
| hsa-miR-1205-R | CCAGTGCAGGGTCCGAGGTA |
| hsa-miR-1205-RT | GTCGTATCCAGTGCAGGGTCCGAGGTATTCGCACTGGATACGACCTCAAA |
| pre-hsa-miR-1205-F | CCGGGAAGGCCTCTGCAGGGTTTGCTTTGAGGTACTTCCTTCCTGTCAACCCTGTTCTGGAGTCTGTTTTTTG |
| pre-hsa-miR-1205-R | AATTCAAAAAACAGACTCCAGAACAGGGTTGACAGGAAGGAAGTACCTCAAAGCAAACCCTGCAGAGGCCTTC |
| E2F1-F | CCGCTCGAGCCGGGGAATGAAGGTGAACA |
| E2F1-R | ATAAGAATGCGGCCGCGAGGCAGGCGCTTCAGAC |
| β-actin-F | TCTGGCACCACACCTTCTAC |
| β-actin-R | CAGCTTCTCCTTAATGTCAC |
| BS2-F(F1) | CTTGAGGGTGGCAGAAGCCAGA |
| BS2-R(R1) | AAGGGTGTCTCCTATCTACAGGCC |
| BS2-F(F2) | CTTGAGGGTGGCAGAAGCCAGA |
| BS2-R(R2) | AAGGGTGTCTCCTATCTACAGGCC |
