# Supplementary material for: Reciprocal regulation of miR-1205 and E2F1 modulates progression of laryngeal squamous cell carcinoma
Source: Cell Death Dis. 2019 Dec 4;10(12):916. doi: 10.1038/s41419-019-2154-4 (PMC6893029; doi:10.1038/s41419-019-2154-4)
Supplement: Supplementary file 2 — Table S2 [file 41419_2019_2154_MOESM2_ESM.docx]

Table S2. Relationship between miR-1205, E2F1 protein expression level and clinicopathologic parameters.

| Characteristics (*n*) | miR-1205 level^a^ | *p*^b^ | E2F1 protein level^c^ | *p*^d^ |
| --- | --- | --- | --- | --- |
| Age |  | 0.6138 |  | 0.8594 |
| <56 (22) | 0.1502 ± 0.1792 |  | 0.5318 ± 0.1604 |  |
| ≥56 (22) | 0.1882 ± 0.2367 |  | 0.5409 ± 0.1775 |  |
| T stage |  | 0.0010 |  | 0.0382 |
| T1-2 (17) | 0.2880 ± 0.2768 |  | 0.4724 ± 0.1986 |  |
| T3-4 (27) | 0.0944 ± 0.0979 |  | 0.5767 ± 0.1328 |  |
| Differentiation |  | 0.3630 |  | 0.0379 |
| Well (16) | 0.1914 ± 0.2081 |  | 0.4644 ± 0.1611 |  |
| Moderately (21) | 0.1205 ± 0.1313 |  | 0.5538 ± 0.1552 |  |
| Poorly (7) | 0.2647 ± 0.3537 |  | 0.6486 ± 0.1604 |  |
| Primary location |  | 0.6312 |  | 0.4466 |
| Supraglottic (15) | 0.1360 ± 0.1527 |  | 0.5620 ± 0.1558 |  |
| Glottic (26) | 0.1902 ± 0.2448 |  | 0.5119 ± 0.1801 |  |
| Subglottic (3) | 0.1532 ± 0.0820 |  | 0.6200 ± 0.0529 |  |
| Lymph node metastasis |  | <0.0001 |  | 0.0063 |
| Negative (26) | 0.2455 ± 0.2427 |  | 0.4850 ± 0.1661 |  |
| Positive (18) | 0.0590 ± 0.0370 |  | 0.6106 ± 0.1425 |  |
| Clinical stage |  | <0.0001 |  | <0.0001 |
| Ⅰ+Ⅱ（12） | 0.3734 ± 0.2895 |  | 0.3758 ± 0.1424 |  |
| Ⅲ+Ⅳ（32） | 0.0927 ± 0.0910 |  | 0.5966 ± 0.1337 |  |

^a^Scores determined by qRT-PCR in mean ± SD.

^b^Mann-Whitney U test (for 2 groups) or Kruskal-Wallis test (for > 2 groups).

^c^Scores determined by western blot in mean ± SD.

^d^Student’s T test (for 2 groups) or one way ANOVA (for > 2 groups).
