# Supplementary material for: Reciprocal regulation of miR-1205 and E2F1 modulates progression of laryngeal squamous cell carcinoma
Source: Cell Death Dis. 2019 Dec 4;10(12):916. doi: 10.1038/s41419-019-2154-4 (PMC6893029; doi:10.1038/s41419-019-2154-4)
Supplement: Supplementary file 3 — Table S3 [file 41419_2019_2154_MOESM3_ESM.docx]

Table S3. Association between the distribution of miR-1205 and E2F1 protein expression in LSCC.

| E2F1 protein | miR-1205 | | Total | *p* |
| --- | --- | --- | --- | --- |
|  | High | Low |  |  |
| High | 7 | 27 | 34 | 0.01 |
| Low | 7 | 3 | 10 |  |
| Total | 14 | 30 | 44 |  |
