# Supplementary material for: Reciprocal regulation of miR-1205 and E2F1 modulates progression of laryngeal squamous cell carcinoma
Source: Cell Death Dis. 2019 Dec 4;10(12):916. doi: 10.1038/s41419-019-2154-4 (PMC6893029; doi:10.1038/s41419-019-2154-4)
Supplement: Supplementary file 4 — Supplementary Figure Legends [file 41419_2019_2154_MOESM4_ESM.docx]

**Fig. S1. MiR-1205 is significantly downregualted in LSCC cells.** (**a**) RT-qPCR analysis of the relative miR-1205 expression in the human LSCC cell lines Hep-2, KB-3-1 and normal bronchial epithelium cell line 16HBE. (**b**) Predicted precursor structure of miR-1205. The E2F1 binding sites are highlighted in blue on the predicted hairpin precursor.

**Fig. S2. Summary of the simulation diagram of reciprocal regulation of miR-1205 and E2F1.**
