# Supplementary figures and images for: Reciprocal regulation of miR-1205 and E2F1 modulates progression of laryngeal squamous cell carcinoma
Source: Cell Death Dis. 2019 Dec 4;10(12):916. doi: 10.1038/s41419-019-2154-4 (PMC6893029; doi:10.1038/s41419-019-2154-4)

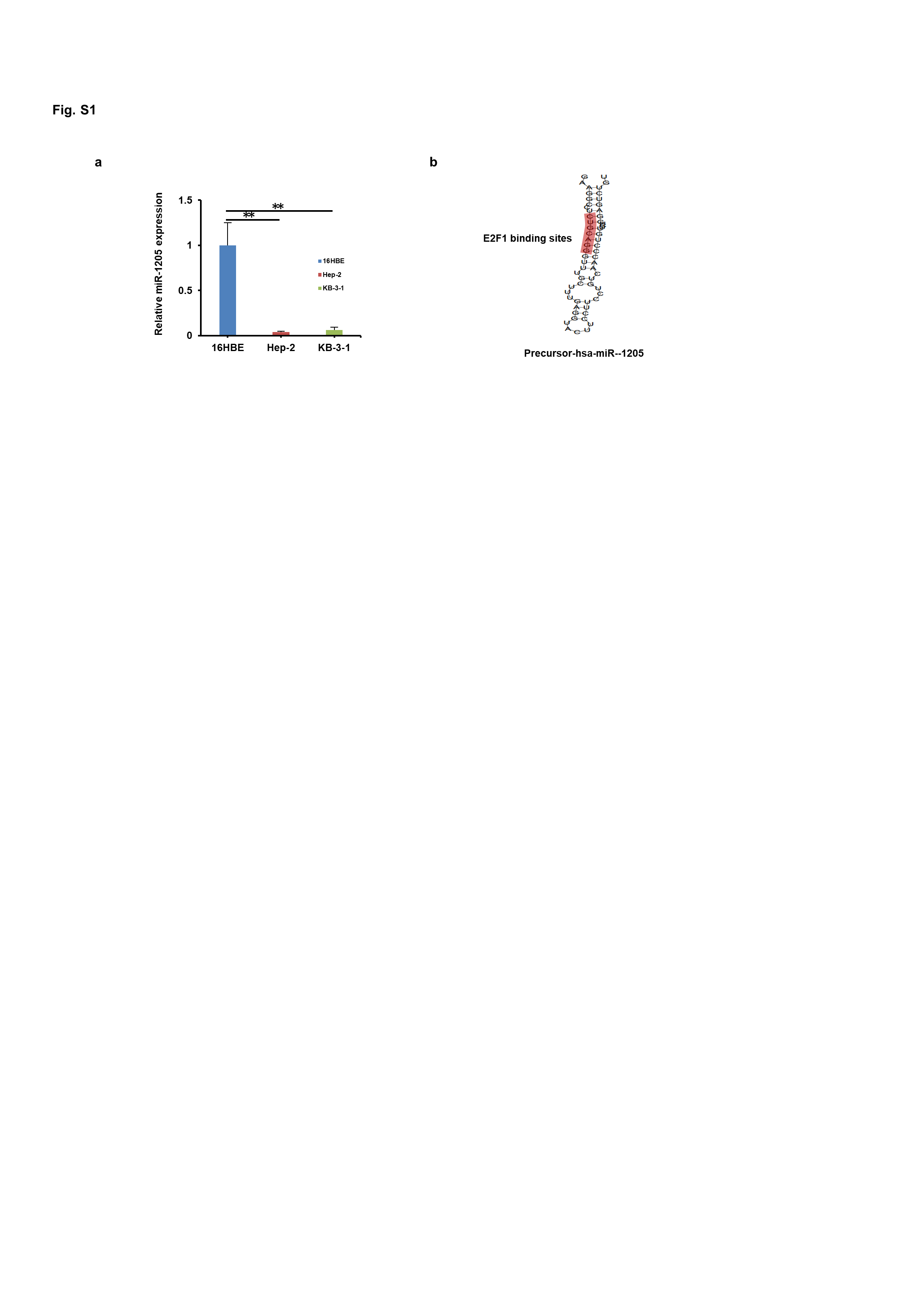

Supplement: Supplementary file 5 — Fig S1 [file 41419_2019_2154_MOESM5_ESM.tif]

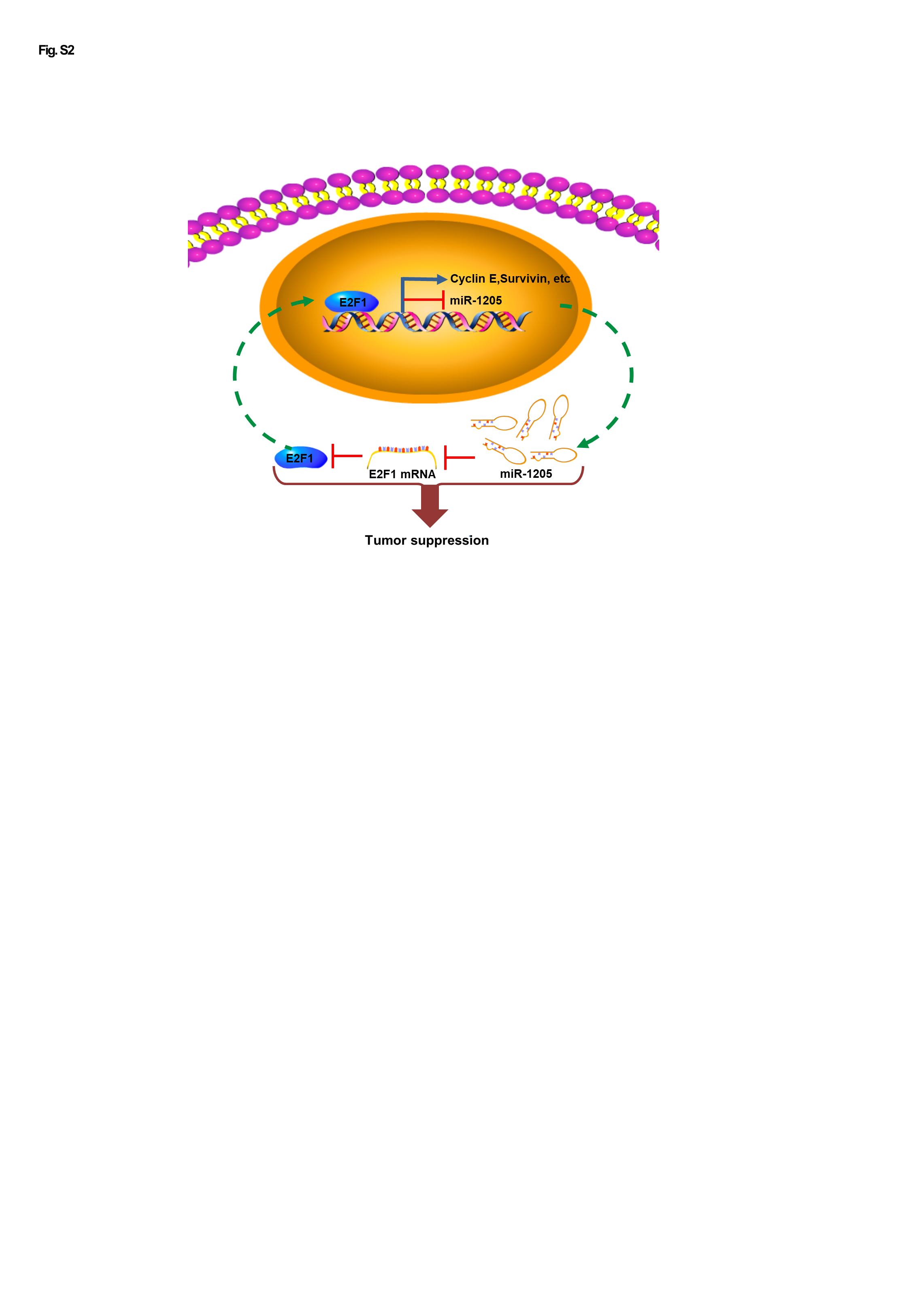

Supplement: Supplementary file 6 — Fig S2 [file 41419_2019_2154_MOESM6_ESM.tif]
